# A preclinical study—systemic evaluation of safety on mesenchymal stem cells derived from human gingiva tissue

**DOI:** 10.1186/s13287-019-1262-5

**Published:** 2019-06-13

**Authors:** Jun Zhao, Julie Wang, Junlong Dang, Wangyu Zhu, Yaqiong Chen, Ximei Zhang, Junliang Xie, Bo Hu, Feng Huang, Baoqing Sun, Joseph A. Bellanti, Song Guo Zheng

**Affiliations:** 1 Department of Clinical Immunology, Third Affiliated Hospital at the Sun Yat-sen University, Guangzhou, China; 2 Division of Immunology and Rheumatology, Department of Internal Medicine, The Ohio State University College of Medicine, Columbus, USA; 3 Division of Rheumatology, Department of Medicine, Milton S. Hershey Medical Center, Hershey, USA; 4 Center of Immunology, Zhoushan City Hospital at Wenzhou Medical University, Wenzhou, China; 5 Department of Laboratory Medicine, Third Affiliated Hospital at the Sun Yat-sen University, Guangzhou, China; 6 Huize Biotech, LLC and Huifu Biotech, LLC, Zhoushan, China; 7 Department of Allergy and Clinical Immunology, State Key Laboratory of Respiratory Disease, National Clinical Research Center of Respiratory Disease, First Affiliated Hospital of Guangzhou Medical University, Guangzhou, China; 8 Department of Pediatrics and Microbiology-Immunology, Georgetown University Medical Center, Washington DC, USA

**Keywords:** Gingival mesenchymal stem cells (GMSCs), Mesenchymal stromal cell, Safety

## Abstract

**Background:**

Mounting evidence has shown that a novel subset of mesenchymal stem cells (MSCs) derived from human gingiva referred to as gingival mesenchymal stem cells (GMSCs) displays a greater immunotherapeutic potential and regenerative repair expression than MSCs obtained from other tissues. However, the safety of the use of transplanted GMSCs in humans remains unclear.

**Methods:**

In this study, we evaluated the safety of GMSCs transplanted into mouse, rat, rabbit, beagle dog, and monkey as well as two animal models of autoimmune diseases.

**Results:**

In short- and long-term toxicity tests, infused GMSCs had no remarkable adverse effects on hematologic and biochemical indexes, particularly on the major organs such as heart, liver, spleen, and kidney in recipient animals. It was also shown that GMSCs were well tolerated in other assays including hemolysis, vascular, and muscular stimulation, as well as systemic anaphylaxis and passive skin Arthus reaction in animal models. GSMC infusion did not cause any notable side effects on animal models of either autoimmune arthritis or lupus. Significantly, GMSCs most likely play no role in genotoxicity and tumorigenesis. The biological features remained stable for an extended period after cell transfer.

**Conclusions:**

GMSCs are safe in various animal models of autoimmunity, even during active disease episodes, especially in monkeys. This study paves a solid road for future clinical trials of GMSCs in patients with autoimmune and inflammatory diseases.

**Electronic supplementary material:**

The online version of this article (10.1186/s13287-019-1262-5) contains supplementary material, which is available to authorized users.

## Background

Recently, mesenchymal stem cells (MSCs) have been considered as a promising therapeutic source for transplantation under various disease settings, especially autoimmune and inflammatory diseases, primarily for their anti-inflammatory and immune-modulatory properties [1–5]. Additionally, MSCs hold great potential for their ability to differentiate into many different tissues and thus are capable of repairing many different damaged tissues [6]. Since the original description of their isolation from the bone marrow by Friedenstein et al. [7], multiple organs and tissues have been reported to be potential sources of MSCs, such as the cord blood [8], umbilical cord [8, 9], adipose tissue [10], amniotic membrane [11], placenta [12], tonsils [13], dental pulp [14], skin [15, 16], and fetal lung and liver [17].

In 2009, Angeles et al. [18] isolated a new population of stem cells from human gingiva, that were referred to as gingiva-derived mesenchymal stem cells (GMSCs), which exhibited clonogenicity, self-renewal, and multipotent differentiation capacities. This stem cell subset not only displayed superior properties of ease of harvesting and expansion in vitro, independency of growth factor and serum requirements, non-tumorigenicity [19], steady phenotype, and telomerase activity in long-term culture but also benefited from additional advantages in addressing ethical concerns of access compared with MSCs isolated from other sources. GMSCs display the same differentiation and regeneration properties with MSCs from other tissues. In vitro, single colony-derived GMSCs have been shown to differentiate into adipocytes and osteoblasts [18]. Using porcine small intestinal submucosa extracellular matrix (SIS-ECM) and human GMSCs as a GMSC/SIS-ECM tissue graft for the tongue reconstruction and the constructs accelerates wound healing and muscle regeneration and maintains the overall tongue shape [20]. In addition, based upon their known immunomodulatory properties, we have previously reported that adoptive transfer of GMSCs in a mouse model of rheumatoid arthritis not only significantly reduced the severity of the arthritis and downregulated the production of Th1 and Th17 inflammatory cytokines (i.e., interferon-γ and interleukin-17A) but also enhanced Treg cell differentiation [3]. We also showed that GMSCs dramatically suppressed contact hypersensitivity by decreasing infiltration of dendritic cells, CD8+ T cells, Th17, and mast cells, as well as a reciprocal increased infiltration of Treg cells [21]. GMSCs co-cultured with macrophages have been shown to convert macrophages into the M2 anti-inflammatory phenotype [6, 22]. In models of experimental colitis and diabetes, systemic infusion of GMSCs significantly ameliorated colonic inflammation, restored the injured gastrointestinal mucosal tissues, and decreased the levels of glucose as well as protected the pathology of the islet in mice [18]. Recently, Huang et al. have shown that GMSCs not only can suppress cell-mediated diseases but also were superior to bone marrow mesenchymal stem cells (BMSCs) in inhibiting xenogenic GVHD in humanized animal models [23, 24]. Collectively, these studies suggest that GMSCs may be a promising candidate for cell-based therapy of autoimmune and inflammatory diseases.

The safety of transplanted GMSCs is an important consideration in the clinical setting. Although, direct endomyocardial transplantation of MSCs has been shown to be safe [25], intravascular infusion may lead to occlusion in the distal microvasculature because of the large cell size [26]. Moreover, long-term transplantation of BMSCs was reported to promote tumor growth [27, 28] and did not achieve effective results for disease treatment [29]. Safety considerations for the therapeutic use of MSCs, therefore, appear to be the main constraint for the development of MSC-based therapy for a variety of human diseases. Until now, there have been no data available that address GMSC clinical safety. The purpose of the present study was to evaluate GMSC safety in various animal strains and models and to use these findings in support of their use in clinical trials.

## Methods

### Cell culture

Human GMSCs were isolated as described previously [18]. Briefly, gingival tissues were obtained from discarded tissues following tooth extraction procedures and were aseptically incubated overnight at 4 °C with dispase (2 mg/ml, Sigma) and then digested at 37 °C for 2 h with 4 mg/ml collagenase IV. The cell suspension was plated on dishes with complete α-MEM (Invitrogen) containing 10% FBS (Gibco), 1% penicillin/streptomycin (Invitrogen), 1% l-glutamine (Invitrogen), and 1% nonessential amino acid (Invitrogen). GMSCs used in the experiments are all third passage with cell purity (CD29 and CD90 expression) at ≥ 95%. Additionally, the differentiation ability of adipogenesis and osteogenesis must be positive and the test for the presence of bacteria, fungus, mycoplasma, hepatitis virus, and endotoxin must all be negative. All individuals provided informed consent for the use of their tissues in this study which was approved by the ethical committee of the Third Affiliated Hospital at the Sun Yat-sen University, China (2018-02-195-01).

### Vascular stimulation test

This experiment is in order to determine whether any substance plays a role in stimulating blood vessels [30]. In this test, New Zealand white rabbits (2.02–2.14 kg) were infused with GMSCs at a dosage of 1.2 × 10^6^/kg via the ear marginal vein and once daily for 2–4 days. A 0.9% NaCl solution was used as a control. Any abnormal changes (such as bleeding, swelling, cyanochroia, or tissue necrosis) occurring in vascular and surrounding tissues were observed to evaluate the influence of GMSCs treatment. After 96 h of the last treatment, vascular and surrounding tissues were collected and fixed with 4% paraformaldehyde and paraffin sections were stained with hematoxylin and eosin (H&E). The infiltration of inflammatory cells and integrity of tissue was analyzed to estimate the degree of vascular irritation.

### Muscle irritation test

A modification of the local muscular tolerance study has been described previously [31]. Briefly, the assay consists of a single intramuscular injection of 1.0 ml GMSC suspension into the right four-headed thigh muscle of rabbits at a concentration of 1.2 × 10^5^ cells/ml. Saline was injected into the corresponding muscle of the left leg as a negative control. At 24 and 48 h after injection, any changes in stimulation such as congestion, edema, degeneration, or necrosis at the injection sites in the muscles were observed. The tissues at the sites of injection were collected and fixed with 4% paraformaldehyde, and paraffin sections were stained with H&E. The infiltration of inflammatory cells and integrity of tissue was analyzed to estimate the degree of muscle irritation.

### Hemolysis test

To detect whether GMSCs could cause lysis of erythrocytes, a hemolytic test was conducted as previously reported [31]. Fresh blood was collected from a healthy rabbit to obtain an erythrocyte suspension. A 2% suspension (2.5 ml) was added to 1.2 × 10^5^/ml of GMSCs from 0.1–0.5 ml; meanwhile, 2.5 ml 0.9% NaCl solution and distilled water was respectively as negative and positive control. The mixed liquids were blended gently and incubated at 37 °C. Hemolysis (supernatant will be red and transparent) or erythrocyte sedimentation was observed at 0.25, 0.5, 0.75, 1, 2, and 3 h after the addition of 2% suspension to GMSCs.

### Systemic anaphylaxis in beagles

Anaphylaxis is a serious allergic reaction triggered by specific antigens that is rapid in onset and may be fatal [32]. In order to investigate the possible effects of GMSCs in systemic anaphylaxis, GMSCs were infused at a dosage of 2 × 10^6^/kg (low-dose group) and 4 × 10^6^/kg (high-dose group) through a forelimb vein, in order to sensitize the models every 2 days for three times. After 14 days of the experiment, a stimulating intravenous injection was applied with a twofold dose that used for sensitization (4 × 10^6^/kg and 8 × 10^6^/kg). A similar volume of 0.9% NaCl solution was used as a control. Changes in the behavior of dogs in each group were observed continuously before and after injection and scored according to the symptoms shown in Additional file 2: Table S1. Moreover, 2 ml of venous blood was obtained from each dog before the first injection for sensitization, before stimulation, and 10 min after the stimulating injection (post stimulation). This blood was centrifuged at 3000 rpm/min for 10 min at room temperature to detect the serum levels of histamine by ELISA kit (TSZ, USA).

### The passive skin Arthus reaction

A passive Arthus reaction was performed as described before [33]. Rat antisera against GMSCs and BSA was prepared as follows: 3.0 × 10^6^/kg (low dose) and 6.0 × 10^6^/kg (high dose) of GMSCs and 100 mg/kg BSA were respectively mixed with complete Freund’s complete adjuvant (1:1) to form emulsion that was subcutaneously injected three times into rats at two sites. GMSCs/BSA were emulsified in incomplete Freund’s adjuvant. This emulsion was administered twice to rats. After 11 days, the serum was collected and was injected into the back of a recipient rat over a surface of about 3 × 4 cm^2^ fur. The same dose of GMSCs, BSA, and 1% Evans’s blue solution was transferred intravenously into rats over a 40-h period. The skin of the Arthus lesion was excised for measurement of the amount of dye leakage after 30 min. A parallel experiment using 0.9% NaCl solution was used to a negative control.

### Short-term and long-term toxicity test

Short-term toxicity tests in rats were performed as follows: 6.0 × 10^7^ and 1.2 × 10^8^/kg of GMSCs was transplanted i.v. into rats, and 0.9% NaCl solution was injected as a negative control. Weight, mortality, reactivity, and histopathological examination were conducted every 24 h for 21 days.

Long-term toxicity test was divided into four groups: (a) negative control 0.9% NaCl solution, (b) low dose 7.5 × 10^6^/kg of GMSCs, (c) middle dose 1.5 × 10^7^/kg of GMSCs, and (d) high dose 3.0 × 10^7^/kg GMSCs. Rats were treated via the tail vein once every 10 days over 30 days. Approximately 2 ml of aortic abdominal blood of each rat was collected before treatment and at the first dose period (FP, 10 days after first dosing), at the withdrawal period (WP, 10 days after the third dosing) and at the recovery period (RP, 4 weeks after withdrawal). The following indices were measured: body weight, food consumption, ophthalmic test and urinalysis, hematological, and biochemical analysis. All blood samples were analyzed by automatic blood cell analyzer (BC-2800, Mindray).

### Toxicity test in autoimmune disease models

The 100 μl serum collected from K/BxN mice, a spontaneous arthritis model, was injected i.p. into C57BL/6 mice (2×). These mice develop a standard K/BxN serum transfer-induced arthritis (STIA) a few days after serum injection as previously reported [34].

A lupus model was established as in our previous report [35]. Briefly, C57BL/6 mouse bone marrow-derived dendritic cells (BMDCs) were prepared and incubated with activated syngeneic lymphocyte-derived DNA (ALD-DNA) for 12 h prior to injection. These cells were then transferred into C57BL/6 mice that will gradually develop lupus syndromes.

2.5 × 10^6^ GMSCs/mouse (which are equivalent to 10-fold higher human clinical dose) were transferred respectively into STIA models via the tail vein and compared with 0.9% NaCl solution. A similar procedure was conducted in the lupus model. Any abnormal reactions such as uneasiness, unsteady standing, or death in GMSC-treated models were observed and hematological and biochemical index was analyzed after 20 days (BC-2800, Mindray).

### *Salmonella typhimurium* reverse mutation assay

This assay is referred to as the “Ames test” that is used as an assay of genotoxicity [36, 37]. Briefly, 100 μl of various concentrations of GMSCs (3.2 × 10^3^, 1.6 × 10^4^, 8.0 × 10^4^, 4.0 × 10^5^, and 2.0 × 10^6^/dish) was separately mixed with 100 μl of *S. typhimurium* mutant strains TA97, TA98, TA102, TA100, and TA1535. Metabolic activity was assayed in the presence of S9 mix, which consists of liver microsomal enzymes S9 (American MOLTOX Corporation), NADP Na_2_, G-6-P-Na, KCl, MgCl_2_, and PBS. These were added to the top agar and sequentially poured into minimal glucose agar plates that were inverted and placed in the dark at 37 °C for 48 h and enumerated for the number of reverse colonies of *S. typhimurium* to determine mutagenesis and genotoxicity of GMSCs. 0.9% NaCl solution was used as a negative control. For positive controls, 50 μg/dish dexon (Sigma) was used to treat TA97, TA98, and TA102 strains, and 2.0 μg/dish NaN3 (Sigma) was used to treat TA100 and TA1535 strains in non-metabolic activity, while in metabolic activity, TA97, TA98, TA100, and TA102 strains were treated with 20 μg/dish 2-aminofluorene (2-AF, Sigma) and TA1535 was treated with 200 μg/dish cyclophosphamide (CP, Sigma).

### Chromosomal aberration assay

In this experiment, Chinese hamster lung fibroblast cell line (CHL), a concentration of 1 × 10^6^ cells/well, was pre-inoculated in a 6-well plate at 37 °C. After 48 h, 2.5 × 10^4^, 5.0 × 10^4^, or 1.0 × 10^5^ of GMSCs was added to the plate and co-cultured in this system. After 6 h, RPMI-1640 complete medium was added to the co-culture system and incubated continuously for 20 h. After this incubation, the system was treated with 0.1 ml colchicine (Sigma, 10 μg/ml) for 4 h and cells were fixed with methanol, acetic acid (3:1), and stained by Giemsa dye for analysis. As a positive control, 0.1 ml mitomycin C (Sigma) in non-metabolic activation and 0.1 ml CP in metabolic activation (presence of 0.5 ml 10% S9 mix) was utilized and 0.9% NaCl solution was used as a negative control [38, 39].

### CM-DiI labeling technique

CM-DI (Life Technologies, 4 μg/ml) was applied to treated GMSCs (2.0 × 10^7^/ml) and incubated at 37 °C for 30 min. Afterward, GMSCs were washed with PBS and finally dispersed in 0.9% NaCl solution. CM-DI-treated GMSCs (7.5 × 10^6^/kg) were transferred into rats through tail vein, with 0.9% NaCl solution injection serving as negative control. On 10, 30, and 58 days, frozen-section samples of the heart, liver, lung, spleen, kidney, brain, thymus, and muscle were prepared, and the distribution and homing of GMSCs in these organs were observed using the fluorescence microscopy (Nikon 80i).

Furthermore, the Cell Tracker Red CMTPX dye (Thermo Fisher Scientific)-labeled GMSCs were infused into C57BL/6 mice. Labeled GMSCs were monitored and sorted for biomarkers (CD90, CD105, CD39, CD73, CD44, HLA-ABC, HLA-DR) by flow cytometry (FACS) on days 1, 2, and 4. To examine the suppressive effect of isolated GMSCs, GMSCs were added to T cells labeled with carboxyfluorescein succinimidyl ester (CFSE, BioLegend) and stimulated with muromonab-CD3ε (0.025 μg/ml) for 3 days in the presence of mitomycin C (BioLegend)-treated antigen-presenting cells (APCs; 1: 1), and proliferation levels were detected with FACS. The GMSC proliferation abilities were tested with CCK-8 kit (Dojindo) according to the manufacturer’s instructions. The survival and apoptosis rate of before and after GMSC injection (day 4) was detected by FACS. The osteogenic and adipogenic differentiation capabilities of sorted GMSCs were also evaluated as follows: cells were plated at 5 × 10^4^ cells/well in 24-well plates overnight and replaced with osteogenic induction medium supplemented with dexamethasone (Sellec), l-glutamine (Gibco), ascorbic acid (Sigma), and β-glycerophosphate (Sigma). For adipogenic differentiation, adipogenic medium containing 10 μM human insulin (Sigma), 1 μM dexamethasone, 200 μM indomethacin (Sigma), and 0.5 mM 3-isobutyl-1-methylxanthine (Sigma) was used. After 4–5 weeks, bone mineralization was assayed by Alizarin Red S (Sigma) staining to detect osteogenic differentiation capabilities of GMSCs, and adipogenic differentiation of GMSCs was assayed by staining with Oil Red O (Sigma) to detect intracellular lipid vacuole characteristic of adipocytes at 2 weeks [18].

### Short-term and long-term toxicity test in rhesus monkeys

In short-term toxicity tests, 5.0 × 10^7^/kg of GMSCs was transplanted i.v. into monkeys, and 0.9% NaCl solution was injected as a negative control. Weight, blood, and biochemical tests were conducted at the time of pre-treatment and at 2, 7, and 15 days following administration. In long-term toxicity tests, 0.3 × 10^7^, 0.6 × 10^7^, and 1.2 × 10^7^/kg of GMSCs, respectively, were transferred into monkeys every 10 days over a 30-day period. Biochemical indices, electrocardiography, ophthalmic testing, and urinalysis were conducted at the first-dose period (FP, 10 days after first dosing), at the withdrawal period (WP, 10 days after the third dosing) and at the recovery period (RP, 6 weeks after withdrawal).

### Statistical analysis

All results were expressed as the mean ± standard deviation. The available data were analyzed using SPSS statistical software version 22.0. Comparisons between groups were performed by analysis of variance (ANOVA) followed by LSD test and Games-Howell test when results from ANOVA were significant. *P* < 0.05 was considered to be statistically significant.

## Results

### GMSCs did not induce inflammatory response in vessel or muscle of rabbit, nor did they give rise to hemolysis in vitro

**Fig. 1:**
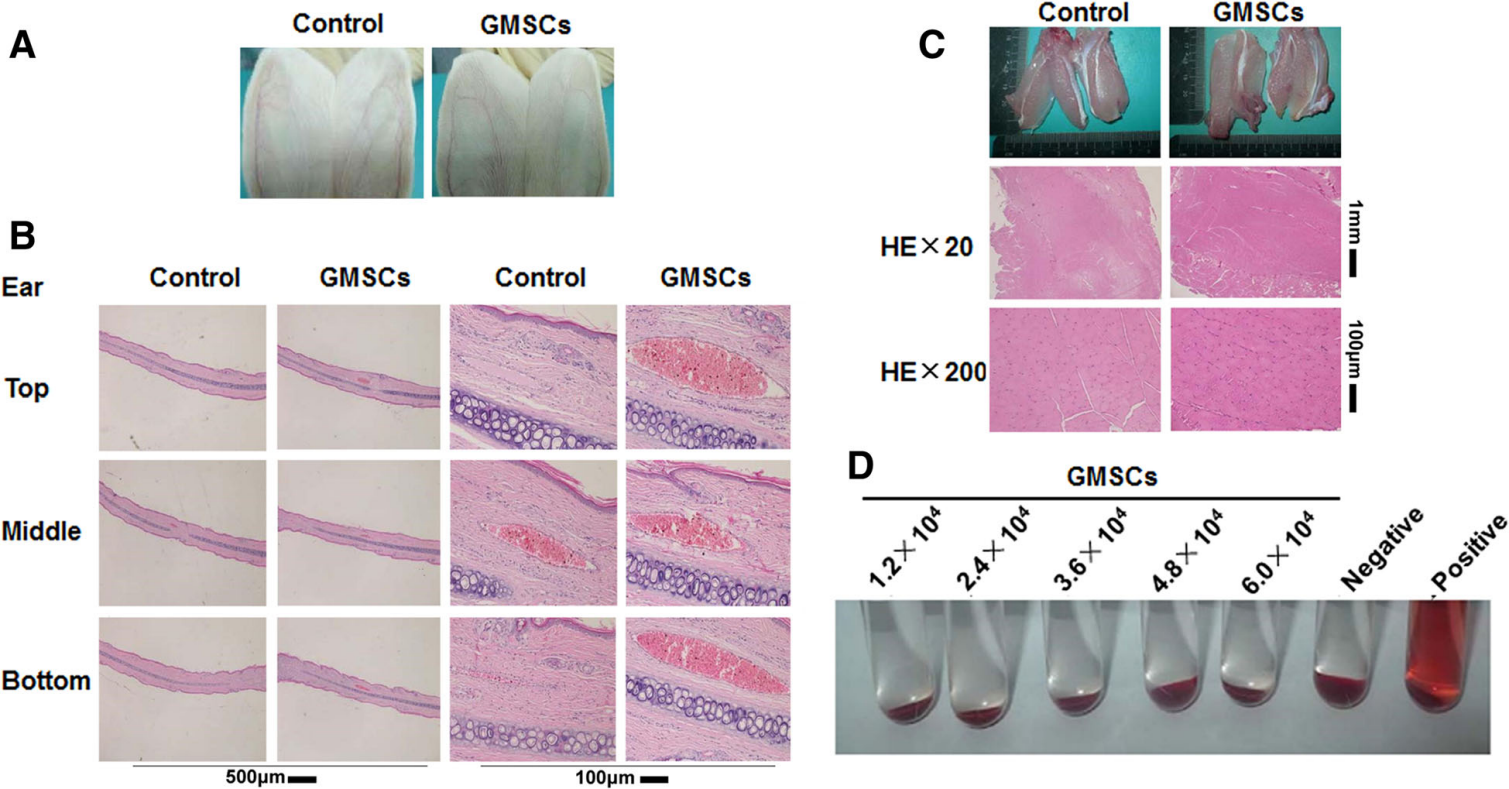
GMSCs do not induce inflammatory responses in blood vessels or muscles and do not induce hemolysis in vitro. **a** Visual inspection of rabbit ear marginal vessel. **b** H&E staining of ear marginal veins in the rabbit. 1.2 × 10^6^/kg of GMSCs were infused into rabbits in 1 ml/side of 0.9% NaCl solution. The same solution was used as a control, *N* ≥ 3 rabbits/group. **c** H&E staining of ear marginal muscle tissues of rabbits infused with 1.2 × 10^5^/ml of GMSCs as before *N* ≥ 6 rabbits/group. **d** Comparison of erythrocytes lysis for each group. A negative control, 0.9% NaCl solution; positive control, distilled water. *N* ≥ 3/group

The results of the vascular stimulation test showed that there was neither evidence of vascular congestion nor swelling of the surrounding tissues in the GMSCs group, mirroring findings similar to those seen in the control group receiving 0.9% NaCl solution (Fig. 1a). H&E staining showed no lymphocytic infiltration in the GMSCs treated group (Fig. 1b). Thus, GMSCs did not cause vascular stimulation.

Local muscular tolerance study was another important toxicity parameter to evaluate the irritation potential of the GMSCs. The results of muscular irritation test are presented as follows: at 24 and 48 h after injection, macroscopic observation, and histopathological examination revealed the horizontal stripes of the muscle fiber to be clear, with no intercellular congestion between muscles nor muscle pathological changes or differences between the GMSCs group and the 0.9% NaCl solution group (Fig. 1c). These results indicate that GMSCs do not cause muscle irritation.

In the hemolysis test, results showed no evidence of hemolysis in either the GMSC group or the negative control group after 3 h of incubation. The positive control group resulted in a uniformly red and transparent color due to the lysed erythrocytes. No erythrocyte sedimentation was observed. This test indicates that the GMSCs had no effect on hemolysis (Fig. 1d).

### GMSCs have no effect on systemic anaphylaxis in beagles and passive anaphylaxis in rats

We next evaluated whether GMSCs could cause a systemic allergic response in a dog model. Our data have demonstrated that beagles exhibited no behavioral changes (including expansion of superficial vessels, such as the mouth, ear, nose, and skin flushing, uneasiness, head hitting against the wall, unsteady standing, falling repeatedly, vomiting repeatedly, salivation, incontinent urination, and defecation) in both low- and high-dose groups that was similar to the 0.9% NaCl solution group (Table 1). Next, we checked the serum levels of histamine and these result indicated that both of low and high dose of GMSCs did not cause increased level of histamine in post-stimulation by contrast to pre-sensitization and pre-stimulation (Additional file 1: Figure S1).

**Table 1:** Judgment of the typical behavior on beagles

Group	Gender	Time course						
		Pre-sensitization	Day 0	Day 2	Day 4	Day 18	Day 19	Day 20
NC	F	**–**	**–**	**–**	**–**	**–**	**–**	**–**
	M	**–**	**–**	**–**	**–**	**–**	**–**	**–**
LD	F	**–**	**–**	**–**	**–**	**–**	**–**	**–**
	M	**–**	**–**	**–**	**–**	**–**	**–**	**–**
HD	F	**–**	**–**	**–**	**–**	**–**	**–**	**–**
	M	**–**	**–**	**–**	**–**	**–**	**–**	**–**

“**–**” represents no symptoms. *NC* negative control, *LD* low dosage group, *HD* high dosage group, *F* female, *M* male. Three times of sensitization was individually conducted in day 0, day 2, and day 4. Stimulation was performed after 14 days (day 18)

**Fig. 2:**
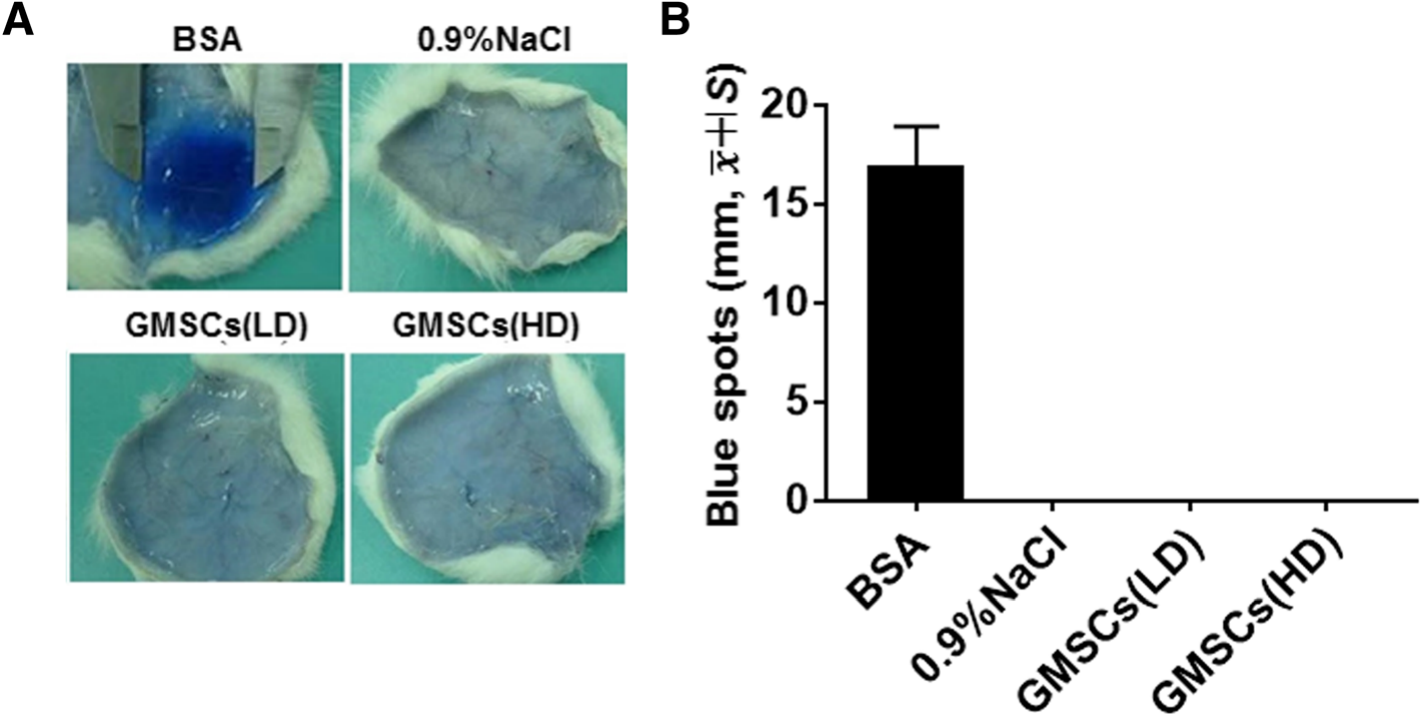
GMSCs have no effect on the passive skin allergy test in rats. BSA (100 mg/kg) was used as a positive control, and 0.9% NaCl solution (5 ml/kg) was used as a negative control. Low dose (LD) of GMSCs was 3.0 × 10^6^/kg. High dose (HD) of GMSCs was 6.0 × 10^6^/kg. *N* ≥ 6 rats/group

Passive anaphylaxis-skin Arthus reaction is a sort of local type III hypersensitivity reaction that are involved in the deposition of antigen (Ag) and antibody (Ab) complexes which mediated acute inflammatory tissue injury [40]. Our data are presented as follows: after 48 h, Evans blue solution was injected intravenously and the amount of dye leakage was measured after 30 min. Although no blue spots in the skin developed in high and low doses of GMSCs (Fig. 2) (*p* < 0.05), cutaneous blue spots were prominently observed in animals in the BSA-treated positive control group. This result demonstrates that GMSCs did not result in the deposition of antigen-antibody complexes to ignite the anaphylaxis-skin Arthus reaction that is a type of local type III hypersensitivity reaction.

### GMSCs have no adverse effects in short- and long-term toxicity test in rats

In order to evaluate the potential toxicity of GMSC administration and to establish an initial dose of usage and the safety spectrum of repeated usage in clinical trials, we next examined the short- and long-term toxicity of GMSCs. Aliquots of 6.0 × 10^7^/kg of GMSCs were injected intravenously into rats (a 60-fold dose relative to patients) who merely appeared with transient decreased light activity and tachypnea at 2 min. In contrast to rats receiving the 1.2 × 10^8^/kg of GMSCs (a dose equivalent with 120-fold of a patient dose), developed decreased activity and shortness of breath over 3 to 5 min. Although 95% rats died, there were no abnormal hematologic or pathologic changes in the important organs and tissues examined in either of the two dosage groups. It is probable that the high dose of GMSCs may have caused an accumulation of GMSCs in the lung resulting in physical death. Importantly, the result of short-term toxicity indicates that 1.2 × 10^8^/kg is the maximum dosage of GMSCs that were used for clinical therapy.

**Fig. 3:**
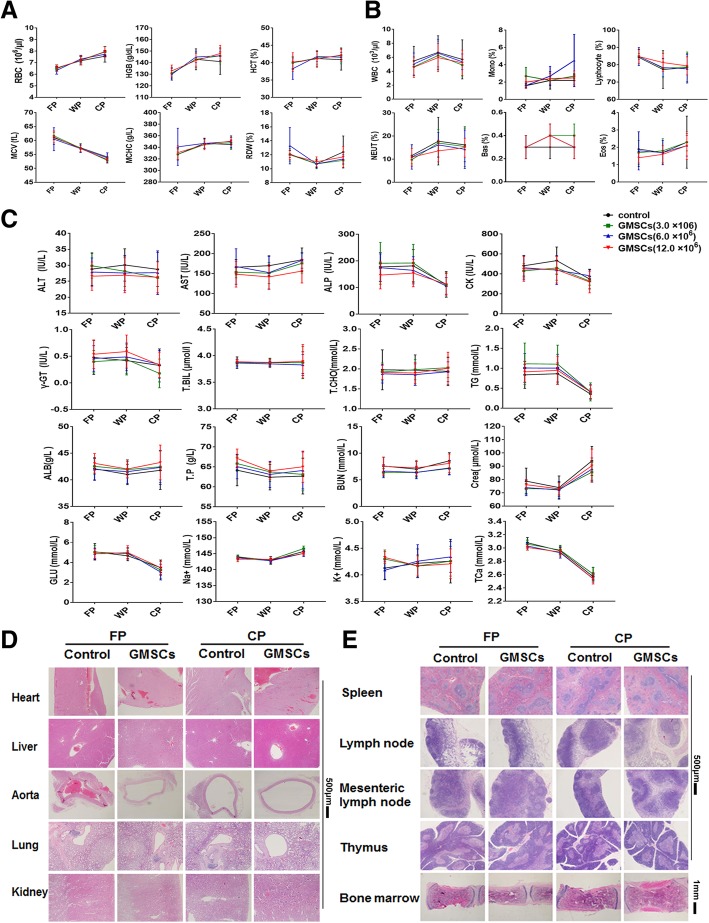
GMSCs have no side effects in long-term toxicity test in rats. **a** GSMC influence on RBC related indexes, including hemoglobin concentration (HGB), hematocrit (HCT), erythrocyte mean corpuscular volume (MCV), mean corpuscular-hemoglobin concentration (MCHC), red blood cell distribution width (RDW), and (**b**) the percentage (%) of immune cells, including white blood cells (WBC), lymphocyte, monocytes (MONO), neutrophilic granulocyte (NEUT), basophilic granulocyte (BASO), eosinophilic granulocyte (EOS) by infusing GMSCs into rats in the first dose period (FP), withdrawal period (WP), and convalescence period (CP) respectively. GMSC effect on biochemical indexes (**c**) and H&E staining (**d**–**e**) of parts of important organ of rats. The data indicate the mean ± SD of three separated experiments. *p < 0.05, **p ≤ 0.01. *N* ≥ 6 rats/group

We next observed the long-term toxicity of GMSC infusion in rats. The weight of rats in the GMSC-treated group was slightly lower, while food consumption, ophthalmic tests, and urinalyses were similar to the control group (data not shown). The results of hematologic analyses are shown in (Fig. 3a, b and Additional file 1: Figure S2A). When infusion of 1.5 × 10^7^ and 3.0 × 10^7^/kg (a dose equivalent to a 15- and 30-fold dose in patients) of GMSCs, mean platelet volume (MPV) and activated partial thrombin time (APTT) in withdrawal period and MPV in recovery period was significantly lower than the control group (*p* ≤ 0.01). Additionally, in the group receiving 3.0 × 10^7^/kg of GMSCs, MPV was also reduced during the first phase of dosing. Moreover, thrombin time (TT) in the 1.5 and 3.0 × 10^7^/kg of GMSC group in WP, and 7.5 × 10^6^/kg (7.5-fold of patient dose) of GMSC group, the percentages of monocytes (MONO) in FP, and basophiles (BASO) in RP were slightly increased in contrast to the control group (*p* ≤ 0.01). A dose-response relationship is observed in all these measurements which conformed to hematologic references. Beyond that, indices of long-term toxicity tests were not different from 0.9% NaCl solution group.

Some biochemical indexes are shown in (Fig. 3c). Both in 0.75 × 10^7^/kg (*p* < 0.05) and 3.0 × 10^7^/kg (*p* ≤ 0.01) of GMSCs, aspartic transaminase (AST) was also decreased in 3.0 × 10^7^/kg GMSC group in withdrawal period. In contrast, concentrations of K^+^ of 0.75 and 3.0 × 10^7^/kg GMSCs and Na^+^ of 0.75 × 10^7^/kg were incremental during the recovery phase in contrast to control group (*p* < 0.05). A dose-response relationship was observed in all these measurements which conformed to biochemical references.

H&E staining (Fig. 3d, e and Additional file 1: Figure S2B) indicated that GMSCs do not have adverse effects on infiltration of inflammatory cells into some important tissue and organs, e.g., heart, liver, spleen, kidney, and thymus. Visceral weights are provided (Additional file 1: Figure S2C). When GMSCs are infused into rats, both the 1.5 and 3.0 × 10^7^/kg dose caused an increase of spleen weight in FP (*p* ≤ 0.01). An increase of heart weight during the first dosing phase was observed in 3.0 × 10^7^/kg group (*p* ≤ 0.01). The 0.75 × 10^7^/kg dose did not result in any organ size abnormalities nor did they produce any side effects. They also demonstrated an excellent long-term (repeated dosing) safety profile.

We also evaluated the safety of GMSC infusion in two autoimmune disease models: autoimmune arthritis and lupus. Our results showed that despite a 10-fold increase in dosage relative to those used in clinics, side effects did not emerge in GMSCs infused models until 20 days after cell injection. Moreover, there were no notable differences in weight, hematological, and biochemical data between the 0.9% NaCl solution group and GMSC group in either autoimmune disease model (Additional file 1: Figure S3). Thus, GMSCs appear to be safe either in health or the disease state.

### GMSCs do not induce reverse mutation of *S. typhimurium* and chromosomal aberration in the CHL line

**Fig. 4:**
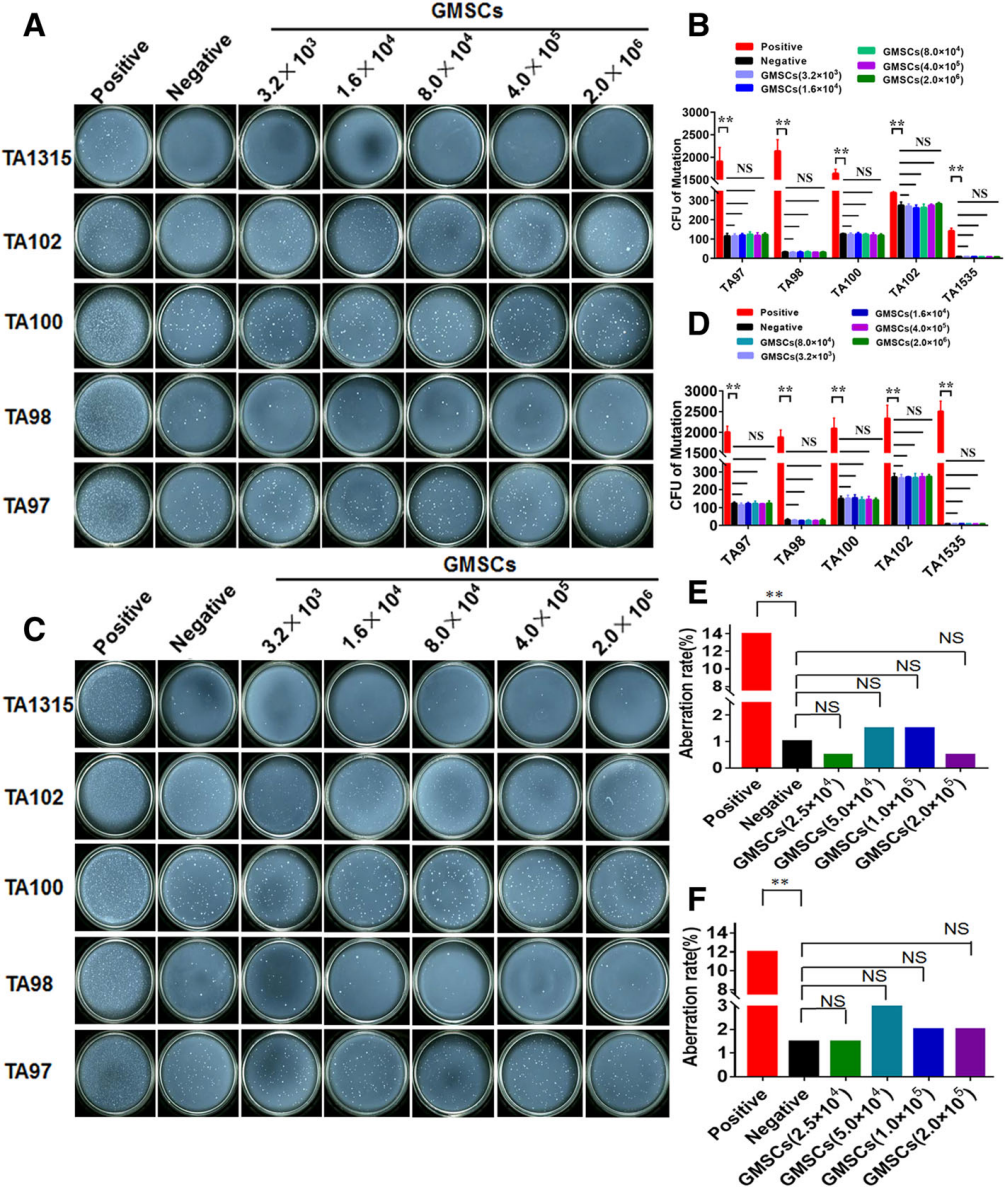
GMSCs did not cause reverse mutation of *S. typhimurium* and chromosome aberration of CHL strain. a, b Under conditions of metabolic activation (presence of S9), positive control (50 μg/dish Dexon was for TA97, TA98, and TA102 strains, 2.0 μg/dish NaN_3_ was for TA100 and TA1535 strains), **c**, **d** non-metabolic activation (absence of S9), positive control (50 μg/dish Dexon was for TA97, TA98, and TA102 stains: 2.0 μg/dish NaN3 was for TA100 and TA1535 stains). Meanwhile, 0.1 ml/dish 0.9% NaCl was as negative control. **e** Chromosome aberration rates under metabolic activation (with S9 mix) and **f** non-metabolic activation (without S9 mix) with 6-h GMSC treatment. CP and mitomycin C served as a positive control with and without S9 mix, and 0.9% NaCl solution was used as a negative control. The data represent the mean ± SD of three separated experiments. NS means no significance; *p < 0.05, **p ≤ 0.01). *N* ≥ 3/group

An Ames test was used in order to determine whether GMSCs resulted in genotoxicity. Figure 4 a and b present the results of metabolic activity as a marker of mutagenesis which requires catalysis by the S9 mix. The results of non-metabolic activity (without S9 mix) are also presented (Fig. 4c, d). These results detect mutagenesis events that can directly induce mutations in *S. typhimurium*. The results were identical between the GMSC group and the negative group and demonstrate that there was no diversification of reverse mutation of colony-forming units detected (*p* ≥ 0.05). The positive control also clearly demonstrates that reverse mutations in colony-forming units were clearly increased (*p* ≤ 0.01) both under conditions of non-metabolic activity and metabolic activity. These data suggest that GMSCs most likely play no role in causing reverse mutation and genotoxicity of *S. typhimurium*.

To evaluate whether GMSCs induce chromosomal aberration in the CHL, various doses of GMSCs were pre-incubated with CHL for 48 h. Under conditions of either metabolic activity or non-metabolic activity conditions, none of the doses of GMSCs had a striking impact on CHL aberration rates (< 5%) relative to the negative group (aberration rates < 5%) over an incubation period of 6 h (*p* ≥ 0.005). Meanwhile, 40 μg/ml of CP and 0.1 μg/ml of mitomycin C (used as a positive control for non-metabolic and metabolic conditions respectively) in this system, aberration rates of CHL were near 10% that was notably higher than negative group (*p* ≤ 0.01) (Fig. 4e, f).

### The distribution and time course of GMSCs in organs of rats

**Fig. 5:**
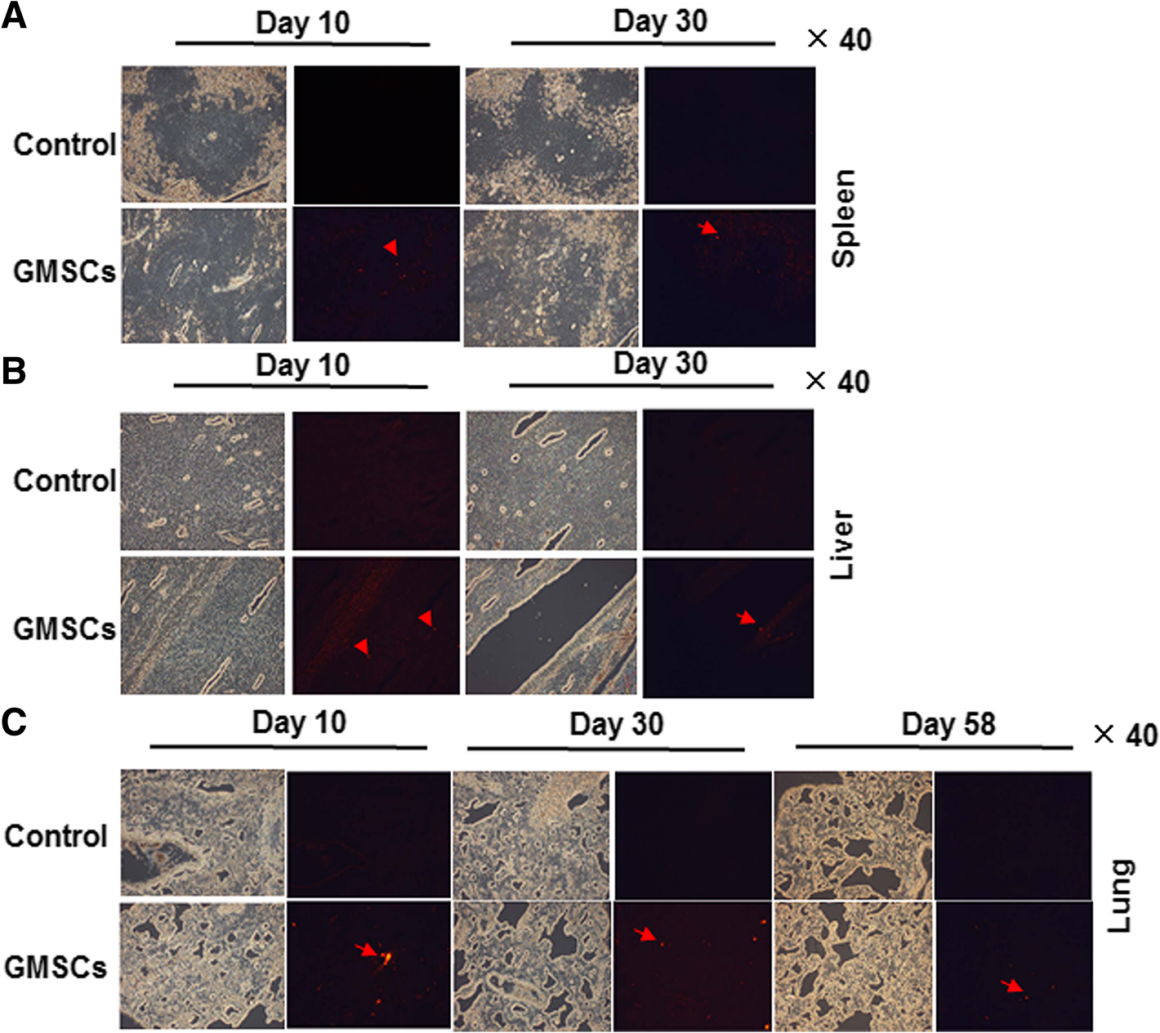
The distribution and time course of GMSCs in organs of rats. **a** The distribution and duration in the spleen, **b** liver, and **c** lung of rats at 10-, 30-, and 58-day time points. Red fluorescence represents CM-DI pre-treated GMSCs (arrowhead). 0.9% NaCl solution-treated rats served as control. *N* ≥ 6 rats/group

In order to determine the GMSCs homing and half-life period in vivo, we transplanted CM-DI pre-treated GMSCs into rats and observed chemotactic effects and distribution of GMSCs. GMSCs were detected only in the spleen (Fig. 5a), liver (Fig. 5b), and lung (Fig. 5c) on days 10 and 30 post-injection. However, it is significant that GMSCs persisted in the lung for 58 days. Nevertheless, fewer GMSCs persisted in other organs and tissues.

We next asked whether the biological characteristics of GMSCs changed following injection into mice. GMSC distribution was monitored on the lung, spleen, lymph nodes, and peripheral blood on days 1 to 4 after injection. As shown in Additional file 1: Figure S4A, GMSCs were primarily found in the lung early and then could be found distributed to the lymph nodes later, indicating that they may have immunoregulatory roles. GMSCs were sorted from the spleen, lymph node, lung, and peripheral blood after they had been transferred. We found that the phenotypes and suppressive activity were nearly identical to that observed prior to injection (Additional file 1: Figure S4B and C). Moreover, there was no change in proliferation abilities of these cells before and after injection (Additional file 1: Figure S4D). We also found that there were no differences in apoptosis rates between sorted GMSCs (4 days after injection) and GMSCs prior to injection, and both cell populations survived well (Additional file 1: Figure S4E). Furthermore, GMSCs that were sorted from mice 4 days after injection maintained osteogenic and adipogenic capacities (Additional file 1: Figure S4F). Thus, GMSCs maintain their phenotype and functional activity in vivo at least during the period of observation used in this study.

### Short-term and long-term toxicity of GMSC infusion in rhesus monkey

Since the study of toxicology is of particular significance in the evaluation of safety of cell-based therapy, and its performance is required for the proposed final product [41], and short-term and long-term toxicity of GMSCs was further conducted in the monkey, a species closer to the human than other animal models. In short-term toxicity tests, 5.0 × 10^7^/kg of GMSCs which is equivalent with 50-fold of clinical dose in patients were transferred to monkeys, and results revealed that GMSCs did not significantly affect the weight of monkeys (*p* ≥ 0.05). Hematologic (Additional file 1: Figure S5A and S5B) and biochemical analyses (Additional file 1: Figure S5C) showed that white blood cells (WBC), neutrophilic granulocyte (NEUT, %), alkaline phosphatase (ALP), and Na^+^ in GMSC-treated group were significantly lower than those in control group at the same points (*p* < 0.05) at the conclusion of the test. However, the γGT and Na^+^ in pre-treated GMSCs (*p* < 0.05) and in the second day of administration (*p* ≤ 0.01) is only slightly higher than the control group. Interestingly, there was no obvious clinical presentation in these monkeys and other indexes were similar to control (*p* ≥ 0.05). Electrocardiogram analysis demonstrated that the heart rate was slightly slower (*p* < 0.05), and S-T segment (*p* ≤ 0.01) and Q-T interval (*p* ≤ 0.01) were more prolonged than the control group when the high dose of GMSCs was infused into monkeys, but still within normal limits and range (Additional file 3: Table S2). Although the Q-T interval delay (*p* ≤ 0.01) contributed to the heart rate, there were no abnormal presentations or death in contrast to the control group.

**Fig. 6:**
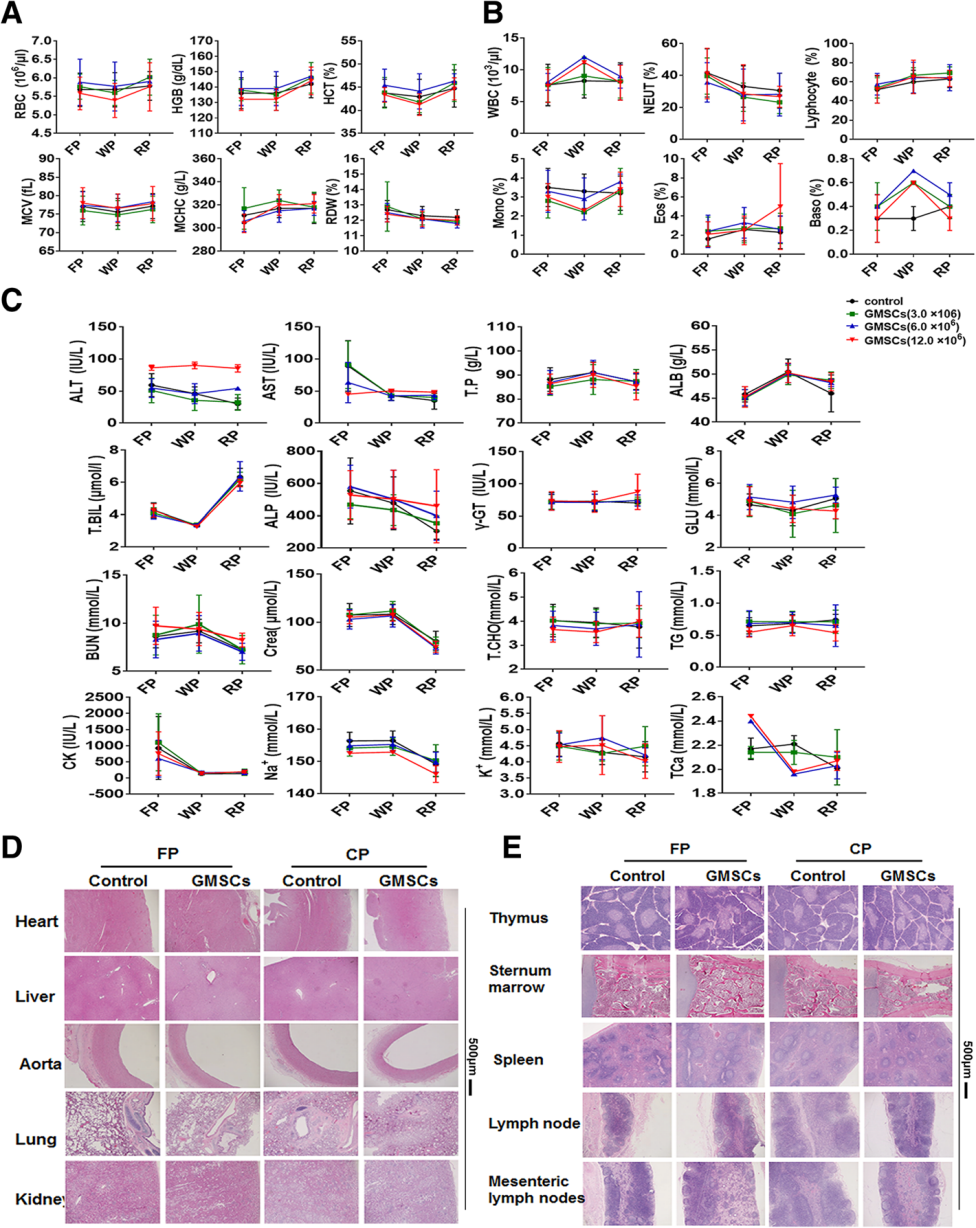
GMSCs do not influence long-term toxicity in rhesus monkeys. **a** Variety of blood indexes including RBC, HGB, HCT, MCV, MCHC, and RDW, and **b** the percentage (%) of immune cells, including WBC, MONO, NEUT, BASO, and EOS, in monkeys during the first-dose period (FP), withdrawal period (WP), and convalescence period (CP), respectively. **c** GMSCs effect on biochemical indexes and H&E staining (**d**, **e**) in an important organ of rhesus monkeys. The data represent the mean ± SD of three separated experiments. *p < 0.05, **p ≤ 0.01. *N* ≥ 10 animals/group

In long-term toxicity tests, the weight (*p* ≥ 0.05), food consumption (*p* ≥ 0.05), bone marrow count (*p* ≥ 0.05), ophthalmic test, and urinalysis of monkeys with the different doses of GMSCs was more aligned with a normal range than that observed for the control group (data not shown). Blood (Fig. 6a, b and Additional file 1: Figure S6A) and biochemical indexes (Fig. 6c) showed that middle and high doses of GMSCs could induce WBC, glutamic-pyruvic transaminase (ALT), Na+, and TCa to increase following the first dose and during the withdrawal period. The BASO (%) during the withdrawal period and the PT in the first-dose period also increased. We also found that MONO (%) decreased during the withdrawal period. We observed that when injected with the middle dose of GMSCs, the following parameters were also influenced: ALT, WBC, BASO (%), lymphocytes (LYMPH, %), MONO (%), and TCa. However, the only changes in blood parameters observed for the low dose of GMSCs were decreased platelet (PLT) and BASO% compared to control. It should be noted that the changes in these indexes were reversible and within normal limits. Despite these observations, H&E staining revealed no differences in important viscera between monkeys pre-treated with GMSCs and those in the control group (Fig. 6d, e and Additional file 1: Figure S6B). While infusion of GMSCs appears to increase spleen weight, it only does so transiently (Additional file 1: Figure S6C). Although the electrocardiographic abnormalities manifested in GMSC treated monkeys resulted in a more prolonged QRS than control animals’ recovery periods (*p* ≤ 0.01) (Additional file 4: Table S3), they were still within the normal range. Other indexes, e.g., morphologic analysis of bone marrow cells (Additional file 5: Table S4) were similar to the control group (*p* ≥ 0.05).

## Discussion

MSCs are immunosuppressive multipotent cells capable of differentiating into many different varieties of specialized cells [42]. Recently, a novel subset of MSCs that originated from human gingival tissue (GMSCs) was found to possess the homologous capacity as other MSCs that maintain immune homeostasis and prevent autoimmunity. GMSCs are particularly effective at suppressing human PBMC-initiated xenogenic responses in a humanized GVHD model [3, 18, 23]. Moreover, GMSCs exhibit a high proliferative rate relative to other adult dental tissues such as dental pulp stem cells (DPSCs), periodontal membrane stem cells PDLSCs, and even BMSCs. Additionally, GMSCs are isolated from human gingiva making them an easily accessible tissue to retrieve from the oral cavity or as a discarded tissue sample following certain dental procedures [18]. These characteristics suggest that GMSCs could represent a novel source of MSCs for the treatment of autoimmune and inflammatory diseases. Despite the advantages of GMSCs in cell therapy, there is an obvious lack of evidence detailing their safety in either animals or humans. Since safety of a cellular product remains the most important criteria for human applications [43, 44], such studies must be evaluated in acute and chronic in vivo models and must encompass [14] the examination of major organs, neighboring tissues, blood chemistry, and blood cell counts after the transplantation into the in vivo models [45].

Quality control (QC) of GMSCs is necessary to be performed prior to their safety evaluation. QC includes cell purity, differentiation activity, and a pathogenic microorganism test in the suspension of GMSCs. Additionally, previous studies have demonstrated that GMSCs consistently express CD29, CD73, and CD90/Thy-1 from passages 2 to 6 and that they can be steadily expanded in vitro while still maintaining their early phenotypes until passage 6 [18]. With this in mind, we opted to use cells at the third passage. It will be interesting to determine whether different passages of GMSCs have different biological features, a notion that richly deserves an independent study in the future.

Previous studies have shown that intravascular infusion of adipose-derived MSCs may lead to occlusion in the distal microvasculature because of the large cell size [26]. In the present study, we firstly provided evidence, under appropriate dosing conditions that GMSC infusion did not result in vascular and muscular stimulation following intravenous and intramuscular administration. GMSCs did not clog in the lung but distributed to lymph nodes and other organs. We are still unclear whether the size difference between different MSC populations will affect their distribution. GMSCs did not induce inflammatory responses at injection sites. We next demonstrated that GMSCs did not destroy erythrocyte integrity by hemolytic testing in vitro. Since injection is the generally acceptable form of administration of GMSCs for cell-based therapy, it is necessary to ensure the safety of various routes of injection before clinical application. Our findings suggest that GMSCs can be applied to multiple diseases through vascular and muscular routes of administration.

Our results also addressed concerns of allergenicity of GMSCs by performance of passive anaphylaxis studies in the rat skin and systemic allergic symptoms in a dog model. We presented evidence that appropriate dose of GMSCs did not cause any passive allergic response, also known as a local type III hypersensitivity reaction. Furthermore, in our experiments conducted in beagles, there were no allergic reactions nor behavioral changes observed, even in high-dose groups. This suggests that GMSCs can safely be used, even in large doses. We will address these issues by the following strategies to avoid this problem: a single dose of GMSCs will be rigorously controlled and autologous GMSCs derived from the patient will be used to provide enhanced safety in therapeutic trials [46].

When GMSCs were administrated in two groups with high doses (mimicking a maximum clinical dose of 60- and 120-fold respectively) for assessment of acute toxicity, we found there were transient and abnormal light responses detected in rats, including decreased activity tachypnea, and weight loss and self-healing in several minutes at low doses. Moreover, 95% death rates occurred in rats at these high doses. This results suggest, therefore, that the optimal maximum tolerated dose is 6.0 × 10^7^/kg in single administration. The long-term view of GMSC administration, suggested that GMSCs had no significant side effects on important organs or tissues, e.g., heart, liver, spleen, lung, and renal function in rats. Other assays, such as ophthalmic test and urinalysis, hematologic, and biochemical indexes, also showed that animals tolerated GMSCs under various conditions. Our studies included both normal animals and those expressing autoimmune diseases. A significant aspect of the toxicology study was that it included non-human primates (NHPs), which are closer to human physiology than other animal models and therefore data generated from NHPs provides more relevant safety data during the preclinical phase. In the studies using NHPs, our data concerning short- and long-term toxicity showed that electrocardiographic, ophthalmic test and urinalysis, hematology, and biochemical indexes of NHPs were all normal when GMSCs were infused in vivo. Collectively, these results suggest that repeated infusion of GMSCs can safely be performed for further research towards in clinical trials.

Tumorigenicity is another important parameter that should be evaluated in the assessment of long-term use of GMSCs. Yamaoka et al. has reported that BMSCs have a tumorigenesis capability, resulting in a long-term risk following the use of BMSCs [19, 28, 47]. BMSCs increase the proliferation, migration, and efficiency of mammosphere formation of tumor cells in vitro and suggested that the promotion of tumor growth in vivo may be also attributable in part to enhanced angiogenesis [48–50]. However, in the present study, we demonstrated GMSCs did not lead to genotoxicity and oncogenesis under therapeutic usage. This result is consistent with the findings of Tomar et al. showing that infusion of GMSCs did not have the tumorigenic potential [18, 51].

Information concerning the kinetics of GMSCs is essential in preclinical studies since kinetics provides reliable evidence for GMSC security and effectiveness, information that is crucial prior to use in clinical research. A report by Li et al. [52] suggests that MSCs are quickly trapped lungs, without any detectable homing to the liver and other organs after inferior vena cava infusion. However, in the present study, preliminarily exploration of chemotaxis and half-life duration in rats demonstrated that GMSCs following intravenous injection mainly distribute to the liver, spleen, and particularly in the lung, with GMSCs persisting for about 2 months. It is not clear why the distribution of GMSCs should be different from other MSCs. These differences merit future study. These findings are consistent with results of other groups that demonstrated systemic delivery of BMSCs was limited by entrapment of cells mainly in the lungs as well as in the liver and spleen [53, 54]. Since infused GMSCs may be localized in the lung and liver in animal models, it is likely that GMSCs have a great potential in the treatment of pulmonary and hepatic diseases.

## Conclusion

In summary, we have conducted a comprehensive evaluation of the safety of GMSCs in this preclinical study. Our evidence strongly suggests that GMSCs have no apparent adverse effects in various animal models including autoimmune arthritis and lupus, particularly in NHPs. Additionally, GMSCs maintain their phenotype, vitality, and function after they are injected into the body. Thus, GMSCs could be a promising therapeutic means for treating many diseases that currently are not curable.

## Additional files

Additional file 1: Supplementary figures and legends. **Figure S1.** The changes of histamine in both low and high dose of GMSC-treated beagle dog groups. **Figure S2.** GMSCs have no side effects in the coagulation system and organs of rats. **Figure S3.** GMSCs have no notable side effects and toxicity in autoimmune disease models. **Figure S4.** GMSCs were recovered from mice. **Figure S5.** GMSCs did not affect short-term toxicity in rhesus monkeys.** Figure S6.** GMSCs have no side effects in the coagulation system and organs of monkeys. (DOCX 3891 kb)

Additional file 2: **Table S1.** Rating criteria of abnormal behavior. (XLS 26 kb)

Additional file 3:
**Table S2.** Electrocardiogram of rhesus by intravenous injection of GMSCs in short-term toxicity test (*n* = 4, $\overline{x}$ ± S). (XLS 29 kb)

Additional file 4:
**Table S3.** Electrocardiogram of rhesus by intravenous injection of GMSCs in long-term toxicity test (*n* = 4, $\overline{x}$ ± S). (XLS 29 kb)

Additional file 5:
**Table S4.** Bone marrow count of rhesus by intravenous injection of GMSCs in long-term toxicity test (*n* = 4, $\overline{x}$ ± S). (XLS 31 kb)

## Abbreviations

2-AF: 2-Aminofluorene
ALP: Alkaline phosphatase
ALT: Glutamic-pyruvic transaminase
APCs: Antigen-presenting cells
APTT: Activated partial thrombin time
AST: Aspartic transaminase
BASO: Basophils
BMSCs: Bone marrow mesenchymal stem cells
CFSE: Carboxyfluorescein succinimidyl ester
CHL: Chinese hamster lung fibroblast cell line
CP: Cyclophosphamide
FP: First-dose period
GLP: Good laboratory practice
GMSCs: Gingival mesenchymal stem cells
H&E: Hematoxylin and eosin
HCT: Hematocrit
HGB: Hemoglobin concentration
LYMPH: Lymphocytes
MCHC: Mean corpuscular-hemoglobin concentration
MCV: Erythrocyte mean corpuscular volume
MONO: Monocytes
MPV: Mean platelet volume
MSCs: Mesenchymal stem cells
NEUT: Neutrophilic granulocyte
NHP: Non-human primate
PLT: Platelet
RDW: Red blood cell distribution width
RP: Recovery period
SIS-ECM: Small intestinal submucosa extracellular matrix
TT: Thrombin time
WP: Withdrawal period
γGT: γ-Glutamyl transpeptidase
