# Supplementary material for: A preclinical study—systemic evaluation of safety on mesenchymal stem cells derived from human gingiva tissue
Source: Stem Cell Res Ther. 2019 Jun 13;10:165. doi: 10.1186/s13287-019-1262-5 (PMC6567625; doi:10.1186/s13287-019-1262-5)
Supplement: Supplementary file 1 — Supplementary figures and legends. Figure S1. The changes of histamine in both low and high dose of GMSC-treated beagle dog groups. Figure S2. GMSCs have no side effects in the coagulation system and organs of rats. Figure S3. GMSCs have no notable side effects and toxicity in autoimmune disease models. Figure S4. GMSCs were recovered from mice. Figure S5. GMSCs did not affect short-term toxicity in rhesus monkeys. Figure S6. GMSCs have no side effects in the coagulation system and organs of monkeys. (DOCX 3891 kb) [file 13287_2019_1262_MOESM1_ESM.docx]

**Supplemental materials**

**
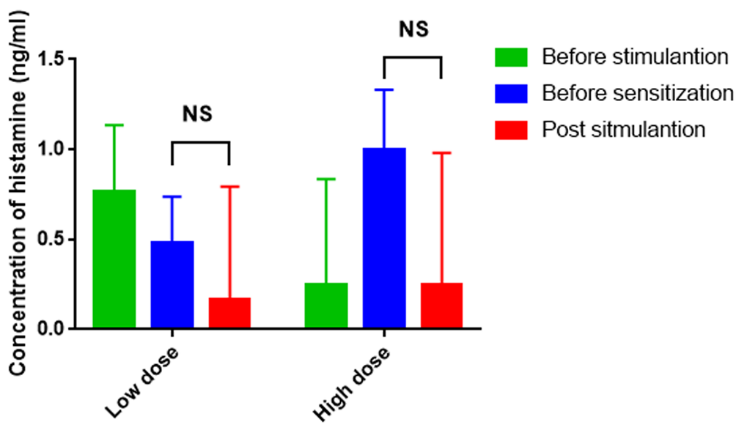
**

**Fig. S1 The changes of histamine in both and high dose of GMSCs treated beagle dog groups.** GMSCs were intravenously injected into beagle dogs as doses of 2×10^6^/kg (low dose) and 4×10^6^/kg (high does) individually for sensitization. After 14 days, the double doses GMSCs were intravenously injected into low dose group (4×10^6^/kg) and high dose group(8×10^6^/kg) individually for stimulation. The serums were collected before sensitization, before stimulation and post stimulation individually. The concentrations of histamine were detected with ELISA. The data indicate the mean ± SD of two independent experiments (NS, not significant).


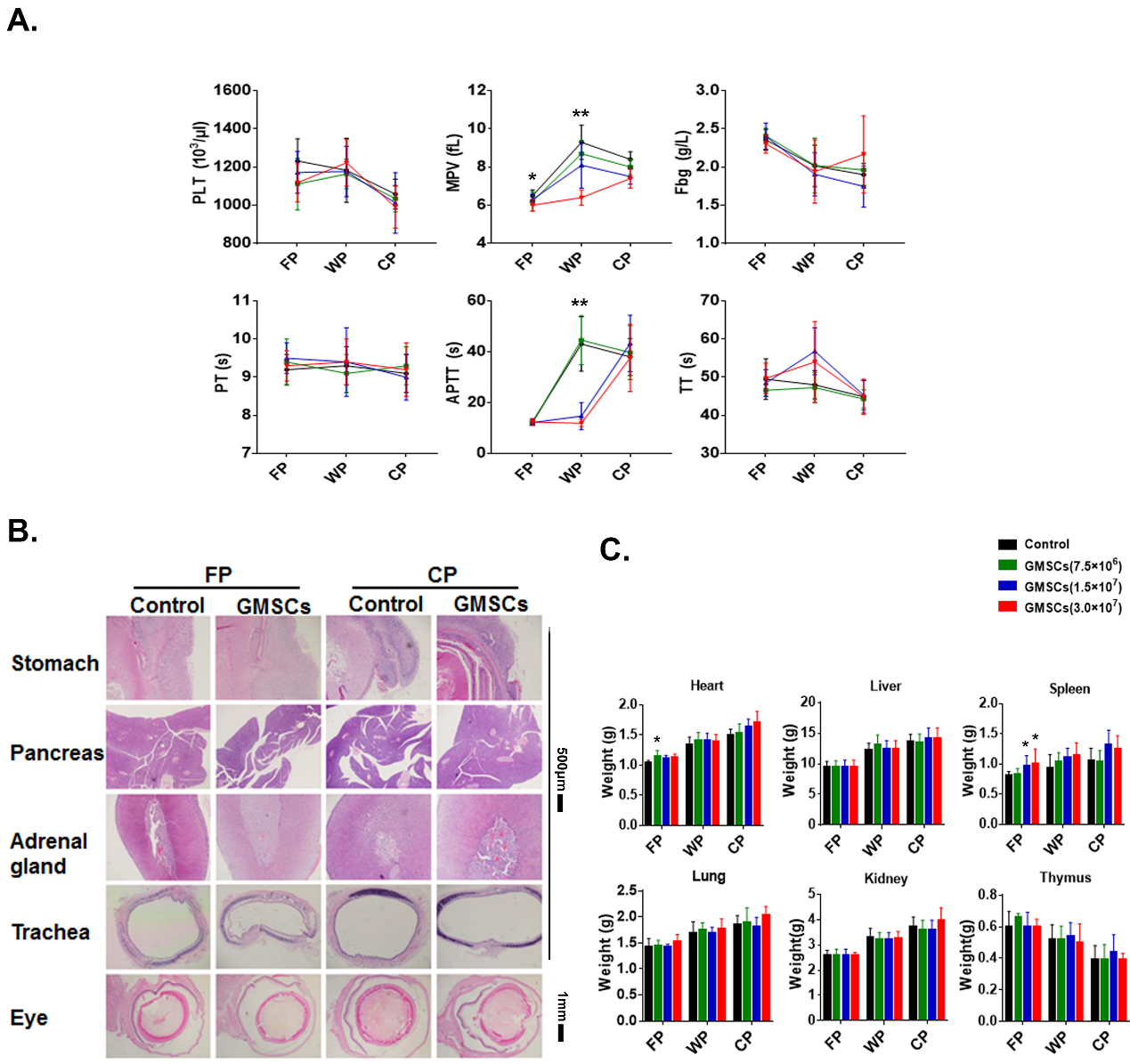


**Fig. S2 GMSCs have no side effects in coagulation system and organs of rats.** (A) Influence of coagulation systemic indexes, including fibrinogen (Fbg), mean platelet volume (MPV), activated partial thrombin time (APTT), Prothrombin time (PT) and thrombin time (TT) by infusing GMSCs into rats in first dose period (FP), withdrawal period (WP) and convalescence period (WP) respectively. (B) H&E staining of parts of secondary organs e.g. stomach pancreas, adrenal gland, trachea and eye. (C) GMSCs effect on weight of some important organs. The data indicate the mean ± SD of three separated experiments, *P<0.05, **P≤0.01. N≥6 rats/group.


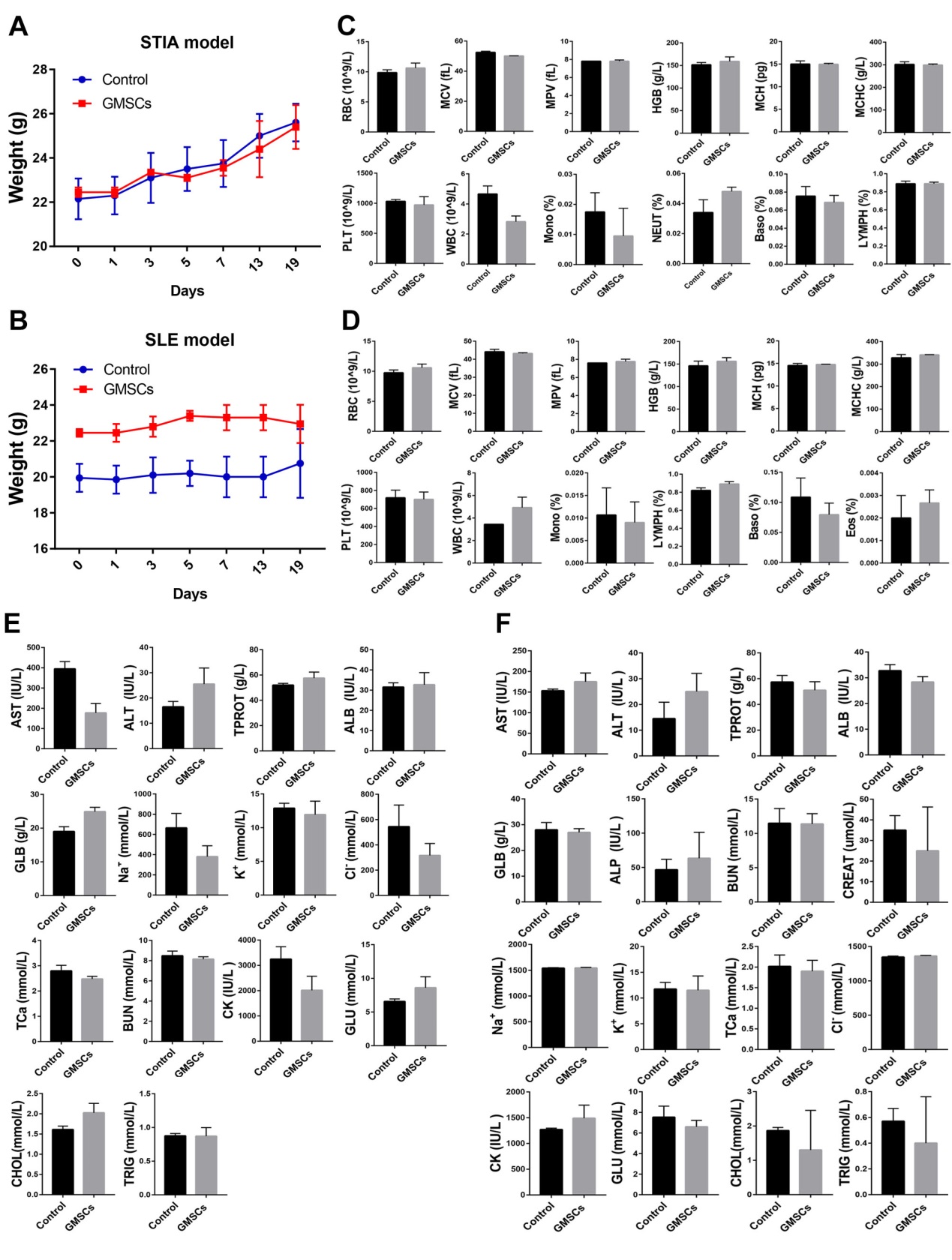


**Fig. S3 GMSCs have no notable side effects and toxicity in autoimmune disease models.** (A, B) the weight of GMSCs treated STIA and lupus models compared with 0.9% NaCl solution group. (C, D) hematological index such as RBC, HGB, HCT, MCV, MCHC, WBC, LYMPH, MONO, NEUTE etc. of STIA and lupus models; (E, F) biochemical index such as AST, ALT, TPROT, ALB, GLB, ALP, K, Na, Cl, GLU, BUN, CREAT and CK etc. of STIA and lupus models. The data indicate the mean ± SD of three separated experiments, *P<0.05, N≥3 animals/experiment.

**
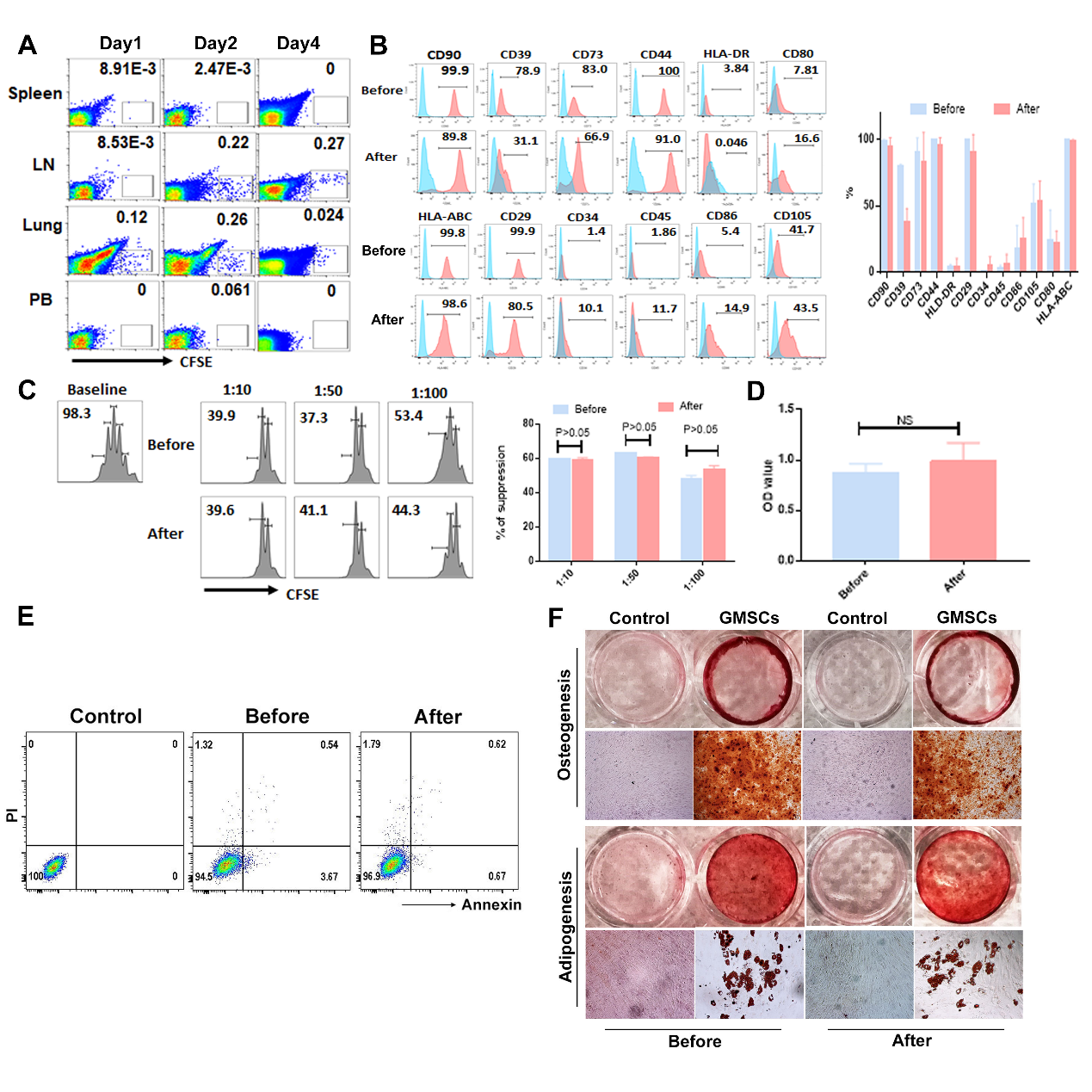
**

**Fig. S4** **GMSCs were recovered from mice.** The CMTPX dye labeled GMSCs were intravenously (3×10^6^/mouse) or intraperitoneally (3×10^6^/mouse) injected into C57BL/6 mice for timing as indicated. The CMPTX labeled GMSCs were then sorted for phenotypes, immunosuppressive potential, proliferation capacities detection, vitality and differentiation. (A) GMSCs distribution in lung, lymph node, spleen and blood was detected by FACS at the day 1, day 2 and day 4. (B) The phenotypes of the sorted GMSCs (24 hours after injection) and the non-injected GMSCs (Before) were detected with FACS. Typical FACS histogram (*left*) and summary data (*right*) were shown. (C) For the immunosuppressive test, T lymphocytes were isolated from C57BL/6 mice, labeled with CFSE and co-cultured with GMSCs in a T cell activation system (the ratio of antigen presenting cells to T cells was 1:1，the concentration of anti-mouse CD3ε was 0.025 μg/ml) for 3 days. The T cell proliferation was detected with FACS. Typical FACS histogram (*left*) and summary data (*right*) were shown. (D) The GMSCs proliferation capacities were detected with cck-8 kit. (E) The survival and apoptosis rate of before and after injected GMSCs (day 4) was detected by FACS. (F) Osteogenic and adipogenic ability of sorted GMSCs from model (4 days after injection) and non-infused GMSCs (Before). The data indicate the mean ± SD of three independent experiments (NS, not significant).


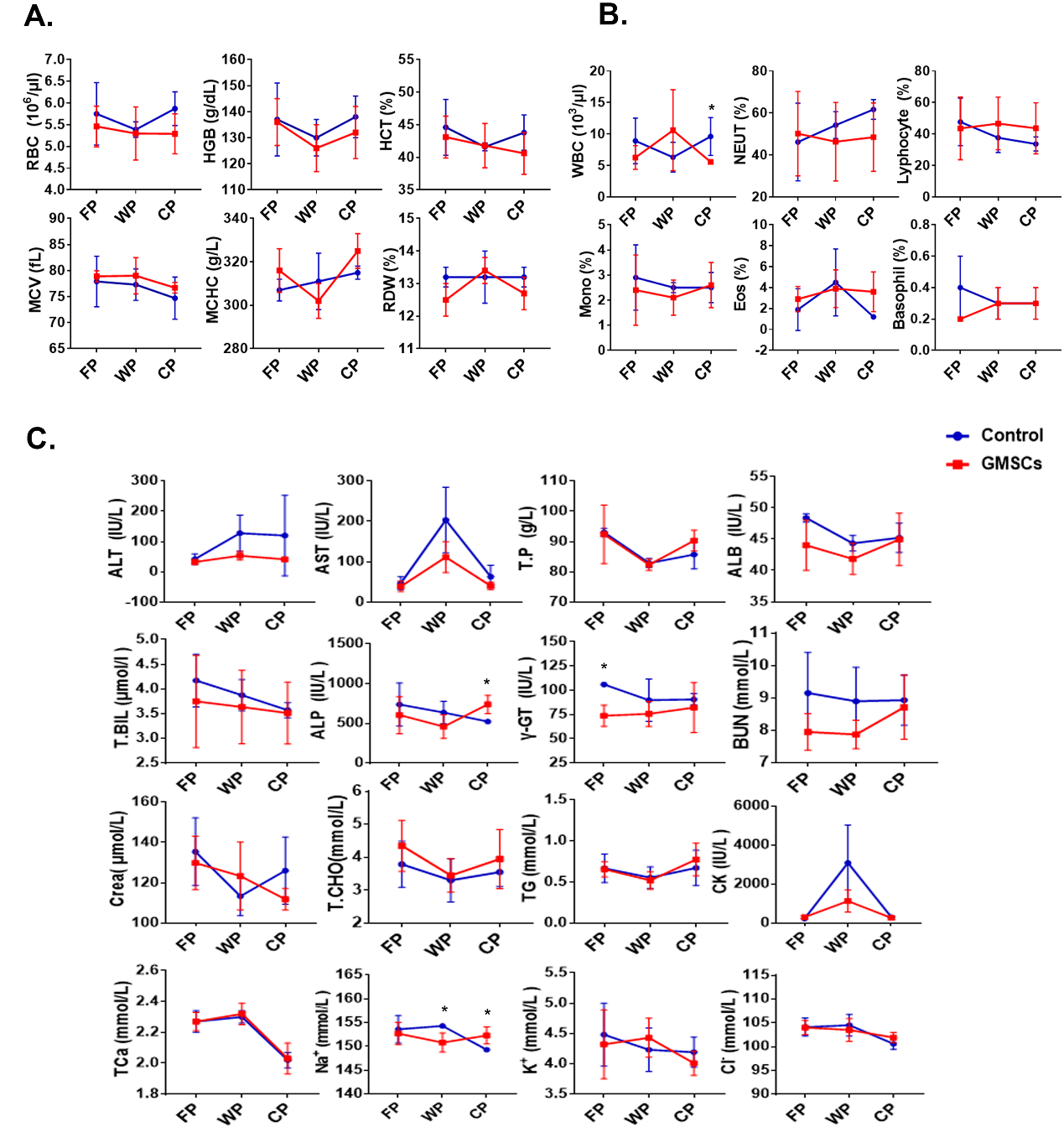


**Fig. S5 GMSCs did not affect short- term toxicity in rhesus monkeys.** (A) variety of blood indexes including RBC, HGB, HCT, MCV, MCHC, RDW, and (B) the percentage (%) of immune cells, including white blood WBC, lymphocyte, MONO, NEUT, BASO, EOS by infusing GMSCs into monkeys in first dose period(FP), withdrawal period (WP) and recovery period (RP) respectively. (C) GMSCs effect on biochemical indexes monkeys. The data indicate the mean ± SD of three separated experiments, *P<0.05, **P≤0.01. N≥4 animals/group.


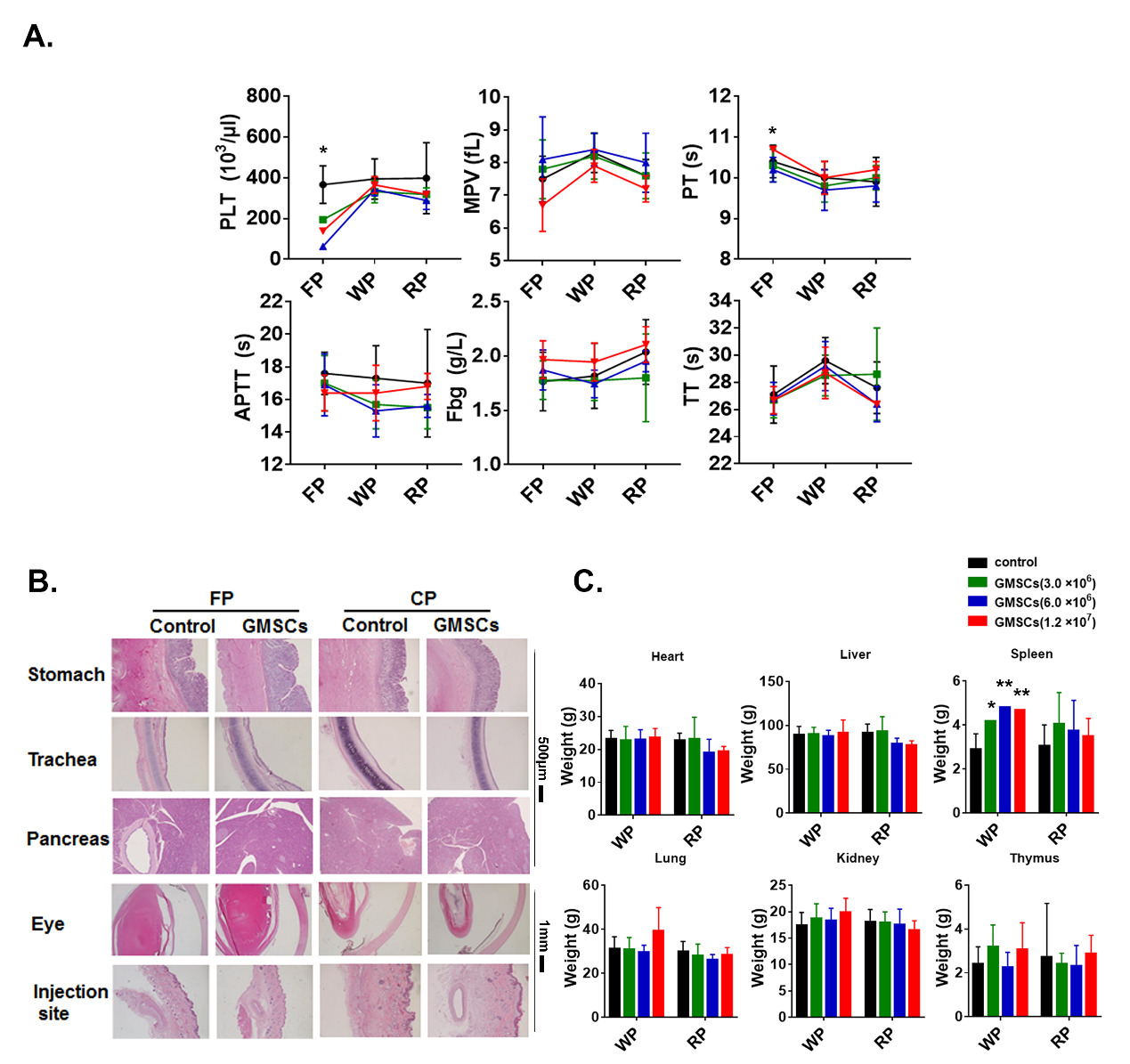


**Fig. S6** **GMSCs have no side effects in coagulation system and organs of monkeys.** (A) Influence of coagulation systemic indexes, including fibrinogen (Fbg), mean platelet volume (MPV), activated partial thrombin time (APTT), Prothrombin time (PT) and thrombin time (TT) by infusing GMSCs into rats in first dose period (FP), withdrawal period (WP) and convalescence period (WP) respectively. (B) H&E staining of parts of secondary organs e.g. stomach pancreas, injection site, trachea and eye. (C) GMSCs effect on weight of some important organs. The data indicate the mean ± SD of three separated experiments, *P<0.05, **P≤0.01. N≥6 rats/group.
